# Supplementary material for: Systematic Review and Meta-Analysis: Prevalence of Non-Alcoholic Fatty Liver Disease and Liver Fibrosis in Patients with Inflammatory Bowel Disease
Source: Nutrients. 2023 Oct 24;15(21):4507. doi: 10.3390/nu15214507 (PMC10648917; doi:10.3390/nu15214507)
Supplement: Supplementary file 1 [file nutrients-15-04507-s001.zip › nutrients-2674742-supplementary-done.pdf]

Prevalence of Non-Alcoholic Fatty Liver Disease and Liver Fibrosis in Patients with Inflammatory Bowel Disease: A Systematic Review and Meta-Analysis

Supplementary Material

Table S1. Search strategies carried out in three bibliographic databases for documents that report on prevalence of non-alcoholic fatty liver disease in patients with inflammatory bowel disease and number of documents retrieved.

Database	Search Strategy	Documents (n)
PubMed	("Fatty Liver"[MeSH Terms] OR "Non-alcoholic Fatty Liver Disease"[MeSH Terms]) AND ("Inflammatory Bowel Diseases"[MeSH Terms] OR "Crohn Disease"[MeSH Terms] OR "colitis, ulcerative"[MeSH Terms]) AND ("Fatty Liver"[MeSH Terms] OR "Non-alcoholic Fatty Liver Disease"[MeSH Terms] OR "metabolic associated fatty liver disease"[All Fields] OR "nonalcoholic steato-hepatitis"[All Fields] OR "hepatic steatosis"[All Fields])	157
Scopus	("fatty liver" OR "Non-alcoholic Fatty Liver Disease" OR "metabolic associated fatty liver disease" OR "nonalcoholic steato-hepatitis" OR "hepatic steatosis") AND ("inflammatory bowel disease" OR “crohn” OR "ulcerative colitis") AND ("incidence" OR "prevalence" OR "epidemiology")	255
Embase	(fatty AND ('liver'/exp OR liver) OR 'non alcoholic') AND fatty AND ('liver'/exp OR liver) AND ('disease'/exp OR disease) AND (inflammatory AND ('bowel'/exp OR bowel) AND ('disease'/exp OR disease) OR crohn* OR ulcerative) AND ('colitis'/exp OR colitis) AND ('incidence'/exp OR incidence OR 'prevalence'/exp OR prevalence OR 'epidemiology'/exp OR epidemiology) AND [embase]/lim	406

Table S2. Characteristics of Studies Involving Prevalence of non-alcoholic fatty liver disease (NAFLD) in patients with inflammatory bowel disease (IBD).

Author. Year	Country	Study Design	Disease Type	IBD Cohort, Mean Age (Range)	Active Disease (%)	IBD Activity Measurement Tool	Diagnostic Method of NAFLD	Mean Age at Diagnosis of NAFLD	Diagnostic Method of Fibrosis
**Full articles**									
Abomhya A, et al. 2022	USA	Retrospective	CD	53 (36-68)	-	-	-	53 (42-63)	-
Arieira C, et al. 2019	Portugal	Cross-sectional	IBD	40.6 ± 12.8	-	-	CAP/HSI/FLI	37.8	-
			CD	-	-	-	CAP/HSI/FLI	-	-
			UC	-	-	-	CAP/HIS/FLI	-	-

Bargiggia S, et al. 2003	Italia	Prospective	IBD	-	61.6	-	US	-	-
			CD	38±14.4	58.5	CDAI	US	-	-
			UC	39±14.4	66.5	-	US	-	-
Bessissow T, et al. 2016	Canada	Retrospective	IBD	33.7 (25.1-46.9)	48	HBI/PMS	HSI	37.7 (27.3-50.1)	FIB-4
Bosch DE, et al. 2017	United States	Retrospective	IBD	47 (43-51)	-	-	US/MRI	49 (44-55)	-
Carrillo-Palau M, et al. 2021	Spain	Cross-sectional	IBD	48 ± 10	-		US	-	TE
			CD	-	11/14	CDAI/HBI	US	-	TE
			UC	-	17	PMS	US	-	TE
Chicco F, et al. 2021	Italy	Prospective	IBD	50	24.6		US	-	-
			CD	48 (34.8-57.5)	15.5	CDAI	US	-	-
			UC	52 (44.5-61.3)	31	PMS	US	-	-
Cohen ME, et al. 2021	United States	Prospective	IBD	14.8 (13 - 16.7)	61	PCDAI/ PUCAI	MRE-PDFF	13.6 (12.5 - 17.1)	MRE-PDFF
Daniluk U, et al. 2021	Poland	Retrospective	IBD	14	97.5	PCDAI/ PUCAI	LFT/US/MRI	-	-
Glassner K, et al. 2017	USA	Retrospective	IBD	19-82	-	-	CT/UC/MRI	45 ± 14.1	-
Hoffmann P, et al. 2020	Germany	Retrospective	IBD	-	74.9		US		-
			CD	-	71.9	HBI	US	49 (20.0)	-
			UC	-	81	SCCAI	US	48.5 (18.5)	-
Hong Q, et al. 2022	China	Retrospective	CD	-	47.4	CDAI	MR-PDFF	32 ± 11	-
Kang MK, et al. 2020	South Korea	Retrospective	IBD	35 (36 - 49.5)	30.25	CDAI/PMS	CT	45.1 ± 13.6	-
Kani HT, et al. 2019	Turkey	Retrospective	IBD	45.59 ± 11.72 (25 - 72)	-	-	CAP	-	-
Koller T, et al. 2017	Slovakia	Prospective	IBD	39 (30.0 - 52.75)	-	-	US	-	-
Li D, et al. 2017	China	Retrospective cases and controls	IBD		-	-	US	-	-
			CD	36.2 ± 12.62	-	-	US	-	-
			UC	48.94 ± 15.26	-	-	US	-	-
Likhitsup A, et al. 2019	USA	Retrospective	IBD	38.6±15	-	-	CT scan	-	-
Magrí S, et al. 2019	Italy	Prospective	IBD	-	27		US	53.9 ± 12.9	TE
			CD	-	42.2	CDAI	US	-	TE
			UC	-	13.7	PMS	US	-	TE
Mancina RM, et al. 2020	Italy	Cross-sectional cases and controls	IBD		17.9		CAP	-	TE
			CD	42.6 ± 13	18.9	HBI	CAP	-	TE
			UC	46.6 ± 11	17.2	PMS	CAP	-	TE
McHenry S, et al. 2020	USA	Retrospective	CD	-	-	-	MR-PDFF	-	FIB-4
Morsy KH, et al. 2012	Egypt	Prospective	UC	31±8 (16-46)	100	Mayo	US+LFT	-	-
Nguyen DL, et al. 2014	USA	Retrospective	IBD		-	-	ICD-9-CM	-	-
			CD	49.38 ± 15.29	-	-	ICD-9-CM	-	-

Author. Year	Country	Study Design	Disease Type	IBD Cohort, Mean Age (Range)	Active Disease (%)	IBD Activity Measurement Tool	Diagnostic Method of NAFLD	Mean Age at Diagnosis of NAFLD	Diagnostic Method of Fibrosis
			UC	48.1 ± 17.45	-	-	ICD-9-CM	-	-
Paparo F, et al. 2012	Italy	Retrospective	CD	50.2±15.9	-	-	CT	-	-
Perrett AD, et al. 1971	UK	Retrospective	UC	12-80	-	-	LFT/Biopsy	-	-
Perrett AD, et al. 1971	UK	Retrospective	CD	16-82	-	-	LFT/Biopsy	-	-
Principi M, et al. 2018	Italy	Retrospective cases and controls	IBD	45.2±15.9	27.5	HBI/PMS	US	49.9±15.8	-
Ritaccio G, et al. 2021	USA	Retrospective	IBD	48±15.6	-	-	Imaging/biopsy	50.6±13.6	NFS
Rodriguez-Duque JC, et al. 2023	Spain	Cross-sectional cases and controls	IBD	52 (19-76)	10.7	HBI/PMS	US	55 (23-76)	TE + Biopsy
Sagami S, et al. 2017	Japan	Retrospective	CD	36.9±12.2	41.6	CDAI	US	42 ± 12.7	-
Saroli Palumbo C, et al. 2019	Canada	Prospective	IBD	42.4	28.6	HBI/PMS	CAP/MRI-PDFF	50.2 ± 12.8	TE/APRI/FIB-4/NFS
Silva J, et al. 2019	Brazil	Cross-sectional	IBD	-	-	-	US/MRI/LFT	-	US/MRI/LFT
Simon TG, et al. 2018	USA	Cross-sectional	CD	40 ± 15	9.7	-	CT	-	FIB-4
Sourianarayanane A, et al. 2013	USA	Retrospective	IBD	-	-	-	US/CT/MRI	46±13.3	-
Spagnuolo R, et al. 2020	Italy	Retrosepective	IBD	44±13	5.6	HBI/Mayo score/ SES-CD	CAP	-	TE
Van Lingen E, et al. 2022	The Netherlands	Prospective	IBD	42.7 (16.0)	31.7	HBI/PMS	CAP	-	TE
Veltkamp C, et al. 2022	Germany	Retrospective	IBD	42 (31-57)	-	-	CAP	45 (40-52)	TE
Voss J, et al. 2021	UK	Cross-sectional	IBD	-	-	-	LFT/ICD-Codes	-	-
			CD	56.6±8.1	-	-	LFT/ICD-Codes	-	-
			UC	57.6±7.9	-	-	LFT/ICD-Codes	-	-
Yamamoto-Furusho JK, et al. 2010	Mexico	Prospective	UC	31.4 ± 13.2	-	-	LFT/US/biopsy	-	Biopsy
Yen H-H, et al. 2021	Taiwan	Retrospective	IBD	37.26 ± 13.16	-	-	CAP	-	LSM
			CD	34.58 ± 12.94	-	-	CAP	-	LSM
			UC	39.4 ± 13.08	-	-	CAP	-	LSM

Author. Year	Country	Study Design	Disease Type	IBD Cohort, Mean Age (Range)	Active Disease (%)	IBD Activity Measurement Tool	Diagnostic Method of NAFLD	Mean Age at Diagnosis of NAFLD	Diagnostic Method of Fibrosis
**Abstracts**									
Almohannadi M, et al. 2020	Qatar	Retrospective	IBD	36.9 ± 13.2	-	-	-	-	-

Andrade P, et al. 2016	Portugal	Retrospective	IBD	43±12	-	-	Biopsy/LFT	-	-
Atanassova A, et al. 2019	Bulgaria	Retrospective	IBD	-			US/LFT	-	-
			CD	-	46.3	CDAI			
			UC	-	43.3	Mayo			
Balaban D. et al. 2017	Romania	Prospective	IBD	45 ± 15			US/HIS/CAP	-	-
			CD	-	51.4	-			
			UC	-	44.4	-			
Basaranoglu M, et al. 2015	Turkey	Retrospective	IBD	-	-	-	US	-	-
Ben Mohamed A, et al. 2019	Tunisia	Retrospective	CD	42.74(18-88)	-	-	US	-	-
Cahill J, et al. 2019	USA	Retrospective	IBD	-	-	-	-	-	-
Cheikhna F, et al. 2020	Morocco	Retrospective	IBD	34.71 (14-82)	-	-	-	-	-
Chhina S, et al. 2014	USA	Retrospective	IBD	-	-	-	US	-	-
Crispino F, et al. 2022	Italy	Retrospective	IBD	46.1±13.4	-	-	HSI/CAP	-	-
Demir M. 2020	Turkey	Retrospective	IBD	44±18	-	-	US	-	FIB-4
Di Girolamo M, et al. 2013	Italy	Retrospective	IBD	-	-	-	US	-	-
Domislovic V, et al. 2019	Croatia	Retrospective	IBD	40	-	-	HSI	-	FIB-4
Dorobăt A, et al. 2018	Romania	Retrospective	IBD	37	-	-	US	-	-
Dundulis J, et al. 2014	USA	Retros pective	IBD	38.6±14.9	-	-	MRI	-	-
Dunn M, et al. 2012	USA	Prospective	IBD	-	-	-	-	-	Biopsy
El Mahjoubi S, et al. 2017	Morocco	Retrospective	IBD	-	-	-	US	-	-
Elchert JA, et al. 2018	USA	Retrospective	CD	-	-	-	-	-	-
Elchert JA, et al. 2018	USA	Retrospective	UC	-	-	-	-	-	-
Erzin Y, et al. 2015	Turkey	Cross-sectional	IBD	44.39±14.1	-	-	US	47.4±13	-
Gilcă-Blanariu G, et al. 2020	Romania	Retrospective	IBD	49.19±14.7	-	-	-	-	-
Gu P, et al. 2022	USA	Retrospective	IBD	52.3±15.22	-	-	CT	-	-
Gupta A, et al. 2022	India	Prospective	UC	29±10.6	-	-	CAP	-	-

Iannone A, et al. 2017	Italy	Retrospective cases-controls	IBD	46.3±15.6	-	-	US	-	TE
Jubin K, et al. 2019	India	Retrospective	IBD	38.3(14-67)	-	-	US	-	TE
Karmiris K, et al. 2014	Greece	Retrospective	IBD	32.9(23-48.4)	-	-	Imaging	-	-
Kasarala G, et al. 2017	USA	Retrospective	IBD	52.13	-	-	ICD-9 Codes	50.55	-
Kumar-Dixit V, et al. 2020	India	Retrospective	IBD	-	-	-	-	-	-
Le NHQ, et al. 2014	USA	Retrospective	IBD	33.8	50	-	HIS/US/CT	40.2	-
Lee A, et al. 2019	USA	Retrospective	IBD	-	-	-	Imaging	-	-
Lee AS, et al. 2022	USA	Retrospective	CD	>18	-	-	MRI	-	FIB-4
Lopes M, et al. 2021	Brazil	Cross-sectional	IBD	45.32±13.59	-	-	US	-	-
Martínez-Domínguez SJ, et al. 2022	Spain	Prospective	IBD	50 (40-60)	10	-	US/CAP	-	TE
Mehrotra P, et al. 2013	India	Prospective	UC	42±4	-	-	US + TE + LFT	-	-
Mehrotra P, et al. 2018	India	Retrospective	UC	-	-	-	TE	-	-
Mohammed T, et al. 2012	Morocco	Retrospective	IBD	37.7 (19-60)	-	-	US	-	-
Monteserin L, et al. 2017	Spain	Retrospective	IBD	47.9±12.54	-	-	US+CAP+HSI+FLI	-	TE
Nasrullayeva F, et al. 2020	Turkey	Retrospective	IBD	44.45±12.71	-	-	LFT + US/CT/MRI	-	-
Patel NP, et al. 2011	India	Prospective	IBD<br>CD<br>UC	-<br>40.6 ± 11.2 (6-82)<br>34.1 ± 14.2 (6-83)	-<br>-<br>-	-<br>-<br>-	CT/MRI/biopsy<br>CT/MRI/biopsy<br>CT/MRI/biopsy	-<br>-<br>-	-<br>-<br>-
Regner E, et al. 2020	USA	Retrospective	IBD	15±2.4 (10-18)	-	-	US/CT/MRI	-	-
Restellini S, et al. 2017	Canada	Prospective	IBD	42.5±15.2	31	HBI/PMS	CAP	-	TE
Sarmini MT, et al. 2019	USA	Retrospective	CD	-	-	-	-	-	-
Sarmini MT, et al. 2019	USA	Retrospective	UC	-	-	-	-	-	-
Scrivo B, et al. 2021	Italy	Prospective	IBD	46.2±15.2	-	-	CAP+HSI	45.4±12.3	CAP
Stoleru G, et al. 2020	USA	Retrospective	IBD	-	-	-	Imaging/biopsy	50.6 ± 13.6	NFS
Sousa P, et al. 2021	Portugal	Retrospective	IBD	46±14	-	-	CAP	-	TE

Thayumanavan JG, et al. 2010	India	Prospective	IBD	31-40	-	-	-	-	-
Thin LW, et al. 2012	Australia	Prospective	IBD	43 ± 15	-	-	TE	-	TE
Tirath A, et al. 2019	India	Prospective	CD	35 (26-49)	-	-	CAP	39 (30-49)	TE
Young S, et al. 2015	USA	Retrospective	CD		-	-	-	-	-

IBD, Inflammatory bowel disease; CD, Crohn disease; UC, ulcerative colitis; NAFLD, Non-alcoholic fatty liver disease; LFT, Liver function tests; US, Ultrasound; CAP, Controlled attenuation parameter; HSI, hepatic steatosis index; FIB-4, fibrosis-4 index; MRI, Magnetic resonance imaging; CT, computed tomography; TE, Transient elastography; ICD codes: International Classification of Diseases codes; FLI, Fatty liver index; HBI, Harvey-Bradshaw Index; CDAI, Crohn disease activity index; NFS, NAFLD fibrosis score; PMS, Partial mayo score; PCDAI, Paediatric Crohn disease activity index; PUCAI, Paediatric ulcerative colitis activity index; MRE-PDFF, Magnetic Resonance Enterography Proton Density Fat Factor; SCCAI, Simple Clinical Colitis Activity Index; APRI, AST-to-Platelet Ratio Index; SES-CD, Simple Endoscopic Score for Crohn disease; LSM, Liver stiffness measure.

Table S3. Prevalence of non-alcoholic fatty liver disease and fibrosis in IBD cohorts.

Author. Year	Disease Type	IBD n	Male n (%)	Female n (%)	NAFLD n (%)	NAFLD Male n (%)	NAFLD Female n (%)	Fibrosis n (%)	MAFLD n (%)
Abomhya A, et al. 2022	CD	215049	90876 (42,3%)	124176 (57,7%)	5268 (2,4%)	2139 (40.6%)	3129 (59.4%)	-	1522 (0.7%)
Almohannadi M, et al. 2020	IBD	913	550 (60.2%)	363 (39.8%)	108 (11.8%)	-	-	-	-
	CD	383	-	-	45 (11.7%)	-	-	-	-
	UC	530	-	-	63 (11.9%)	-	-	-	-
Andrade P, et al. 2016	IBD	54	35 (64.8%)	19 (35.2%)	13 (24.1%)	-	-	-	-
Ariera C, et al. 2019	IBD	161	75 (46.6%)	86 (53.4%)	73 (45.3%)	42 (57.5%)	31 (42.5%)	-	21 (13%)
Atanassova A, et al. 2019	IBD	320	-	-	178 (55.6%)	-	-	-	-
	CD	160	-	-	95 (59.4%)	-	-	-	-
	UC	160	-	-	83 (51.9%)	-	-	-	-
Balaban D, et al. 2017	IBD	62	31 (50%)	31 (50%)	23 (37.1%)	-	-	-	-
Bargiggia S, et al. 2003	IBD	511	263 (51.5%)	248 (48.5%)	194 (37.9%)	-	-	-	-
	CD	311	144 (46.3%)	167 (53.7%)	123 (39.5%)	-	-	-	-
	UC	200	119 (59.5%)	81 (40.5%)	71 (35.5%)	-	-	-	-
Basaranoglu M, et al. 2015	IBD	2700	1593 (59%)	1107 (41%)	1080 (40%)	-	-	-	-
Ben Mohamed A, et al. 2019	CD	86	42 (48.8%)	44 (51.2%)	21 (24.4%)	7 (33.3%)	14 (66.6%)	-	-
Bessissow T, et al. 2016	IBD	321	151 (47%)	170 (53%)	108 (33.6%)	47 (43.5%)	61 (56.5%)	7 (6.4%)	9 (2.8%)
	CD	217	-	-	81 (37.3%)	-	-	-	-
	UC	104	-	-	27 (26%)	-	-	-	-
Bosch DE, et al. 2017	IBD	93	-	-	34 (36.6%)	14 (48.3%)	15 (51.7%)	-	6 (6.4%)
	CD	39	-	-	24 (61.5%)	-	-	-	-
	UC	54	-	-	10 (18.5%)	-	-	-	-
Cahill J, et al. 2019	IBD	1186	573 (48.3%)	613 (51.7%)	151 (12.7%)	71 (47%)	80 (53%)	-	79 (6.7%)
Carrillo-Palau M, et al. 2021	IBD	136	-	-	63 (46.3%)	-	-	-	-

Cheikhna F, et al. 2020	IBD	218	110 (50.5%)	108 (49.5%)	8 (3.7%)	-	-	-	-
Chhina S, et al. 2014	IBD	1304	-	-	126 (9.7%)	-	-	-	-
Chicco F, et al. 2021	IBD	142	76 (53.5%)	66 (46.5%)	58 (40.8%)	-	-	-	-
	CD	58	36 (62.1%)	22 (37.9%)	27 (46.6%)	-	-	-	-
	UC	84	40 (47.6%)	44 (52.4%)	31 (36.9%)	-	-	-	-
Cohen ME, et al. 2021	IBD	83	50 (60.2%)	33 (39.8%)	5 (6%)	4 (80%)	1 (20%)	0 (0%)	2 (2.4%)
	CD	57	-	-	4 (7%)	-	-	-	-
	UC	23	-	-	0	-	-	-	-
Crispino F, et al. 2022	IBD	227	110 (48.5%)	117 (51.5%)	71 (31.3%)	-	-	-	-
Daniluk U, et al. 2021	IBD	119	69 (58%)	50 (42%)	4 (3.4%)	-	-	-	-
	CD	42	-	-	1 (2.4%)	-	-	-	-
	UC	77	-	-	3 (3.9%)	-	-	-	-
Demir M. 2020	IBD	539	280 (51.9%)	259 (48.1%)	172 (31.9%)	-	-	21 (12.2%)	-
Di Girolamo M, et al. 2013	IBD	788	-	-	128 (16.2%)	-	-	-	-
Domislovic V, et al. 2019	IBD	250	130 (52%)	120 (48%)	91 (36.4%)	-	-	9 (9.9%)	-
	CD	167			61 (36.5%)	-	-	5 (8.2%)	-
	UC	83			30 (36.1%)	-	-	4 (13.3%)	-
Dorobăț A, et al. 2018	IBD	135	80 (59.3%)	55 (40.7%)	27 (20%)	-	-	-	16 (11.9%)
	CD	38			7 (18.4%)	-	-	-	-
	UC	97			20 (20.6%)	-	-	-	-
Dundulis J, et al. 2014	IBD	70	31 (44.3%)	39 (55.7%)	29 (41.4%)	-	-	-	8 (11.4%)
Dunn M, et al. 2012	IBD	1463	-	-	15 (1%)*	-	-	-	-
	CD	873	-	-	9 (1%)*	-	-	-	-
	UC	590	-	-	6 (1%)*	-	-	-	-
El Mahjoubi S, et al. 2017	IBD	113	-	-	20 (17.7%)	-	-	-	-
Elchert JA, et al. 2018	CD	153810	-	-	520 (0.3%)	-	-	-	468 (0.3%)
Elchert JA, et al. 2018	UC	129300	-	-	370 (0.3%)	-	-	-	359 (0.3%)
Erzin Y, et al. 2015	IBD	276	132 (47.8%)	144 (52.2%)	100 (36.2%)	56 (56%)	44 (44%)	-	-
	CD	104	-	-	43 (41.3%)	-	-	-	-
	UC	172	-	-	57 (33.1%)	-	-	-	-
Gilcă-Blanariu G, et al. 2020	IBD	384	226 (58.9%)	158 (41.1%)	108 (28.1%)	-	-	-	-
	CD	118	-	-	34 (28.8%)	-	-	-	-
	UC	266	-	-	74 (32.7%)	-	-	-	-
Glassner K, et al. 2017	IBD	421	-	-	56 (13.3%)	-	-	-	23 (5.5%)
Gu P, et al. 2022	IBD	256	145 (56.6%)	111 (43.4%)	27 (10.5%)	-	-	-	-
Gupta A, et al. 2022	UC	107	60 (56.1%)	47 (43.9%)	29 (27.1%)	-	-	-	-
Hoffmann P, et al. 2020	IBD	455	226 (49.7%)	229 (50.3%)	213 (46.8)	115 (54%)	98 (46%)	-	13 (2.9%)
	CD	302	149 (49.3%)	153 (50.7%)	145 (48%)	75 (51.7%)	70 (48.3%)	-	7 (2.3%)
	UC	153	77 (50.3%)	76 (49.7%)	68 (15%)	40 (58.8%)	28 (41.2%)	-	6 (3.9%)
Hong Q, et al. 2022	CD	340	242 (71.2%)	98 (28.8%)	83 (24.4%)	64 (77.1%)	19 (22.9%)	-	29 (8.5%)

Iannone A, et al. 2017	IBD	378	-	-	106 (28%)	-	-	-	-
Jubin K, et al. 2019	IBD	35	18 (51.4%)	17 (48.6%)	8 (22.9%)	-	-	-	-
	CD	18	-	-	6 (33.3%)	-	-	-	-
	UC	17	-	-	2 (11.8%)	-	-	-	-
Kang MK, et al. 2020	IBD	443	284 (64.1%)	159 (35.9%)	49 (11.1%)	31 (63.3%)	18 (36.7%)	-	18 (4.1%)
	CD	274	-	-	20 (7.3%)	-	-	-	-
	UC	169	-	-	29 (23.1%)	-	-	-	-
Kani HT, et al. 2019	IBD	99	58 (58.6%)	41 (41.4%)	44 (44.4%)	-	-	-	-
	CD	58	-	-	28 (48.3%)	-	-	-	-
	UC	39	-	-	15 (38.5%)	-	-	-	-
Karmiris K, et al. 2014	IBD	1489	-	-	159 (10.7%)	-	-	-	-
Kasarala G, et al. 2017	IBD	552887	235351 (42.6%)	317536 (57.4%)	5589 (1%)	2202 (39.4%)	3387 (60.6%)		1246 (0.2%)
	CD	352396	-	-	3767 (1.1%)	-	-	-	-
	UC	200491	-	-	1822 (0.9%)	-	-	-	-
Koller T, et al. 2017	IBD	155	-	-	34 (21.9%)	-	-	-	-
Kumar-Dixit V, et al. 2020	IBD	198	70 (35.4%)	128 (64.6%)	19 (9.6%)	-	-	-	-
	CD	58			4 (6.9%)				
	UC	140	-	-	15 (10.7%)	-	-	-	-
Le NHQ, et al. 2014	IBD	232	116 (50%)	116 (50%)	95 (40.9%)	-	-	-	-
Lee A, et al. 2019	IBD	168	-	-	47 (28%)	-	-	-	-
Lee AS, et al. 2022	CD	46	-	-	17 (37%)	-	-	2 (11.8%)	-
Li D, et al. 2017	IBD	206	133 (64.6%)	73 (35.4%)	22 (10.7%)	-	-	-	-
	CD	137	90 (65.7%)	47 (34.3%)	15 (10.9%)	-	-	-	-
	UC	69	43 (62.3%)	26 (37.7%)	7 (10.1%)	-	-	-	-
Likhitsup A, et al. 2019	IBD	70	-	-	31 (44.3%)	-	-	-	-
Lopes M, et al. 2021	IBD	71	26 (36.6%)	45 (63.4%)	32 (45.1%)	-	-	-	-
Magrì S, et al. 2019	IBD	178	97 (54.5%)	81 (45.5%)	72 (40.4%)	51 (70.8%)	21 (29.2%)	21 (29.2%)	22 (12.4%)
Mancina RM, et al. 2020	IBD	95	57 (60%)	38 (40%)	68 (75.6%)	-	-	-	-
	CD	37	24 (64.9%)	13 (54.2%)	22 (59.5%)	-	-	-	-
	UC	58	33 (56.9%)	25 (43.1%)	46 (79.3%)	-	-	-	-
Martínez-Domínguez SJ, et al. 2022	IBD	700	350 (50%)	350 (50%)	298 (42.6%)	-	-	51 (17.1%)	-
McHenry S, et al. 2020	CD	311	155 (49.8%)	156 (50.2%)	118 (37.9%)	55 (46.6%)	63 (53.4%)	38 (32.2%)	77 (24.8%)
Mehrotra P, et al. 2013	UC	20	14 (70%)	6 (30%)	3 (15%)	-	-	-	-
Mehrotra P, et al. 2018	UC	128	-	-	23 (18%)	-	-	-	-
Mohammed T, et al. 2012	IBD	199	61 (30.7%)	138 (69.3%)	23 (11.6%)	-	-	-	-
Monteserin L, et al. 2017	IBD	88	41 (46.6%)	47 (53.4%)	33 (37.5%)	-	-	14 (42.4%)	-
Morsy KH, et al. 2012	UC	33	15 (45.5%)	18 (54.5%)	15 (45.5%)	-	-	-	-
Nasrullayeva F, et al. 2020	IBD	470	260 (55.3%)	210 (44.7%)	179 (38.1%)	-	-	-	-
	CD	264	-	-	102 (38.6%)	-	-	-	-

	UC	201	-	-	75 (37.3%)	-	-	-	-
Nguyen DL, et al. 2014	IBD	15284	8450 (55.3%)	6834 (44.7%)	2462 (16.1%)	-	-	-	-
	CD	6850	3349 (48.9%)	3501 (51.1%)	1391 (20.3%)	-	-	-	-
	UC	8434	5101 (60.5%)	3333 (39.5%)	1071 (12.7%)	-	-	-	-
Paparo F, et al. 2012	CD	221	114 (51.6%)	107 (48.4%)	24 (10.9%)	-	-	-	-
Patel NP, et al. 2011	IBD	806	490 (60.8%)	296 (36.7%)	49 (6.1%)	-	-	-	-
	CD	162	88 (54.3%)	74 (45.7%)	14 (8.6%)	-	-	-	-
	UC	644	402 (62.4%)	242 (37.6%)	35 (5.4%)	-	-	-	-
Perrett AD, et al. 1971	UC	50	-	-	19 (38%)	-	-	-	-
Perrett AD, et al. 1971	CD	39	-	-	8 (20.5%)	-	-	-	-
Principi M, et al. 2018	IBD	465	241 (51.8%)	224 (48.2%)	130 (28%)	70 (53.8%)	60 (46.2%)	-	18 (3.8%)
	CD	258	-	-	63 (24.4%)	-	-	-	-
	UC	207	-	-	67 (32.4%)	-	-	-	-
Regner E, et al. 2020	IBD	203	-	-	6 (3%)	-	-	-	4 (2%)
	CD	140	-	-	4 (2.9%)	-	-	-	3 (2.1%)
	UC	51	-	-	2 (3.9%)	-	-	-	1 (2%)
Restellini S, et al. 2017	IBD	349	170 (48.7%)	179 (51.2%)	135 (38.7%)	-	-	33 (24.4%)	-
Ritaccio G, et al. 2021	IBD	1672	752 (45%)	920 (55%)	207 (12.4%)	96 (46.4%)	111 (53.6%)	6 (4.3%)	121 (7.2%)
	CD	897	-	-	131 (14.6%)	-	-	-	-
	UC	453	-	-	39 (8.6%)	-	-	-	-
Rodríguez-Duque JC, et al. 2023	IBD	831	401 (48.2%)	430 (51.8%)	349 (42%)*	188 (53.9%)	161 (49.1%)	33 (9.5%)	349 (42%)
	CD	389	-	-	166 (42.7%)*	-	-	-	166 (42.7%)
	UC	420	-	-	174 (41.4%)*	-	-	-	174 (41.4%)
Sagami S, et al. 2017	CD	303	226 (74.6%)	77 (25.4%)	66 (21.8%)	53 (80.3%)	13 (19.7%)	-	24 (7.9%)
Salori Palumbo C, et al. 2019	IBD	384	172 (44.8%)	212 (55.2%)	126 (32.8%)	65 (51.6%)	61 (48.4%)	31 (24.6%)	13 (3.4%)
	CD	248	-	-	78 (31.5%)	-	-	-	-
	UC	136	-	-	48 (35.3%)	-	-	-	-
Sarmini MT, et al. 2019	CD	159290	-	-	4190 (2.6%)	1564 (37.3%)	2626 (62.7%)	-	2030 (1.3%)
Sarmini MT, et al. 2019	UC	125380	-	-	2450 (2%)	1051 (42.9%)	1399 (57.1%)	-	1201 (0,9%)
Scrivo B, et al. 2021	IBD	231	138 (59.7%)	93 (40.3%)	50 (21.6%)	-	-	-	-
Silva J, et al. 2019	IBD	306	117 (38.2%)	189 (61.8%)	22 (7.2%)	-	-	-	-
	CD	141	-	-	11 (7.8%)	-	-	-	-
	UC	165	-	-	11 (6.7%)	-	-	-	-
Simon TG, et al. 2018	CD	462	216 (46.8%)	246 (53.2%)	242 (52.4%)	-	-	32 (13.2%)	-
Sourianarayanane A, et al. 2013	IBD	928	-	-	76 (8.2%)	-	-	-	39 (4.2%)
Sousa P, et al. 2021	IBD	115	48 (41.7%)	67 (58.3%)	55 (47.8%)	-	-	13 (23.6%)	-
Spagnuolo R, et al. 2020	IBD	89	56 (62.9%)	33 (37.1%)	59 (66.3%)	-	-	-	-
Stoleru G, et al. 2020	IBD	1673	-	-	208 (12.4%)	-	-	6 (2.9%)	3 (0.2%)

Thayumanavan JG, et al. 2010	IBD	39	11 (28.2%)	28 (71.8%)	9 (23.1%)	-	-	-	-
Thin LW, et al. 2012	IBD	110	52 (47.3%)	58 (52.7%)	7 (6.4%)	-	-	-	-
Tirath A, et al. 2019	CD	100	63 (63%)	37 (37%)	42 (42%)	33 (78.6%)	9 (21.4%)	6 (14.3%)	5 (5%)
Van Lingen E, et al. 2022	IBD	82	46 (56.1%)	36 (43.9%)	32 (39%)	-	-	16 (50%)	-
Veltkamp C, et al. 2022	IBD	132	57 (43.2%)	75 (56.8%)	40 (30.3%)	17 (42.5%)	23 (57.5%)	7 (17.5%)	-
	CD	79	31 (39.2%)	48 (60.8%)	27 (34.2%)	-	-	-	-
	UC	53	40 (75.5%)	13 (24,5%)	13 (24.5%)	-	-	-	-
Voss J, et al. 2021	IBD	5422	-	-	11 (0.2%)	-	-	-	-
	CD	1738	747 (43%)	991 (57%)	5 (0.3%)	-	-	-	-
	UC	3684	1916 (52%)	1768 (48%)	6 (0.2%)	-	-	-	-
Yamamoto-Furusho JK, et al. 2010	UC	200	106 (53%)	94 (47%)	50 (25%)	-	-	1 (2%)	-
Yen H-H, et al. 2021	IBD	81	58 (71.6%)	23 (28.4%)	24 (29.6%)	18 (75%)	6 (25%)	5 (20.8%)	18 (22.2%)
	CD	36	28 (77.8%)	8 (22.2%)	11 (30.6%)	-	-	-	-
	UC	45	30 (66.7%)	15 (33.3%)	13 (29.9%)	-	-	-	-
Young S, et al. 2015	CD	846	-	-	86 (10.2%)	-	-	-	-

n, number of patients; IBD, inflammatory bowel disease; NAFLD, Non-alcoholic fatty liver disease; MAFLD, Metabolic associated fatty liver disease; CD, Crohn´s disease; UC, Ulcerative colitis.

Table S4. Joanna Briggs Institute critical appraisal checklist for studies reporting prevalence data applied for included studies in the systematic review.

Author. year	1. Was the sample frame appropriate to address the target population?	2. Were study participants sampled in an appropriate way?	3. Was the sample size adequate?	4. Were the study subjects and the setting described in detail?	5. Was the data analysis conducted with sufficient coverage of the identified sample?	6. Were valid methods used for the identification of the condition?	7. Was the condition measured in a standard, reliable way for all participants?	8. Was there appropriate statistical analysis?	9. Was the response rate adequate, and if not, was the low response rate managed appropriately?
Abomhya A, et al. 2022	Yes	Yes	Yes	Yes	Yes	Unclear	Unclear	Yes	NA
Almohannadi M, et al. 2020	Yes	Unclear	Yes	No	Yes	No	Unclear	Yes	NA
Andrade P, et al. 2016	Unclear	Unclear	No	No	Yes	No	Unclear	Yes	NA
Areira C, et al. 2019	Unclear	Unclear	Unclear	Yes	Yes	Yes	Yes	Yes	NA
Atanassova A,et al. 2019	Unclear	Unclear	Yes	No	Yes	Unclear	Yes	Yes	NA
Balaban D, et al. 2017	Unclear	Yes	No	Unclear	Yes	Yes	Yes	Yes	NA
Bargiggia S, et al. 2003	No	Unclear	Yes	No	Yes	Yes	Yes	Yes	NA
Basaranoglu M, et al. 2015	Unclear	Unclear	Yes	No	Yes	Unclear	Unclear	Yes	NA
Ben Mohamed A, et al. 2019	Yes	Unclear	No	No	Yes	Unclear	Yes	Yes	NA
Bessissow T, et al. 2016	No	Yes	Yes	Yes	Yes	Yes	Unclear	Yes	NA
Bosch DE, et al. 2017	No	Yes	No	Yes	Yes	Yes	Yes	Yes	NA
Cahill J, et al. 2019	Yes	Unclear	Yes	Yes	Yes	Unclear	Yes	Yes	NA
Carrillo-Palau M, et al. 2021	No	Yes	Unclear	Yes	Yes	Yes	Yes	Yes	NA
Cheikhna F, et al. 2020	Unclear	Unclear	Yes	No	Yes	Unclear	Unclear	Yes	NA
Chhina S, et al. 2014	Unclear	Yes	Yes	No	Yes	Unclear	Unclear	Yes	NA
Chicco F, et al. 2021	Yes	Yes	Unclear	Yes	Yes	Yes	Yes	Yes	NA
Cohen ME, et al. 2021	Unclear	Yes	No	Yes	Yes	Yes	Yes	Yes	NA
Crispino F, et al. 2022	Unclear	Yes	Yes	No	Yes	Yes	Unclear	Yes	NA
Daniluk U, et al. 2021	Yes	Yes	Unclear	Yes	Yes	Unclear	Yes	Yes	NA
Demir M. 2020	Yes	Yes	Yes	Unclear	Yes	Unclear	Yes	Yes	NA
Di Girolamo M, et al. 2013	Yes	Yes	Unclear	No	Yes	Unclear	Yes	Yes	NA
Domislovic V, et al. 2019	Unclear	Unclear	Unclear	Unclear	Yes	Yes	Yes	Yes	NA
Dorobăț A, et al. 2018	Unclear	Unclear	Unclear	No	Yes	Unclear	Yes	Yes	NA
Dundulis J, et al. 2014	Yes	Yes	No	Unclear	Yes	Unclear	Yes	Yes	NA
Dunn M, et al. 2012	Unclear	Yes	Yes	Unclear	Yes	Unclear	Unclear	Yes	NA
El Mahjoubi S, et al. 2017	Unclear	Yes	No	No	Yes	Unclear	Yes	Yes	NA
Elchert JA, et al. 2018	Unclear	Yes	Yes	Unclear	Yes	Unclear	Yes	Yes	NA
Elchert JA, et al. 2018	Unclear	Yes	Yes	Unclear	Yes	Unclear	Yes	Yes	NA
Erzin Y, et al. 2015	Unclear	Yes	Yes	Unclear	Yes	Unclear	Yes	Yes	NA
Gilcă-Blanariu G, et al. 2020	Yes	Yes	Yes	Unclear	Yes	Unclear	Unclear	Yes	NA
Glassner K, et al. 2017	Unclear	Unclear	Yes	Yes	Yes	Unclear	No	Yes	NA
Gu P, et al. 2022	Unclear	Yes	Yes	Yes	Yes	Unclear	Yes	Yes	NA

Gupta A, et al. 2022	Yes	Yes	Unclear	No	Yes	Yes	Yes	Yes	NA
Hoffmann P, et al. 2020	Unclear	Yes	Yes	Yes	Yes	Unclear	Yes	Yes	NA
Hong Q, et al. 2022	Unclear	Yes	Yes	Yes	Yes	Yes	Yes	Yes	NA
Iannone A, et al. 2017	Yes	Yes	Yes	Yes	Yes	Unclear	Yes	Yes	NA
Jubin K, et al. 2019	Yes	Yes	No	Unclear	Yes	Unclear	Yes	Yes	NA
Kang MK, et al. 2020	Unclear	Yes	Yes	Yes	Yes	Unclear	Yes	Yes	NA
Kani HT, et al. 2019	Unclear	Yes	Unclear	Unclear	Yes	Yes	Yes	Yes	NA
Karmiris K, et al. 2014	Unclear	Unclear	Yes	No	Yes	Unclear	Yes	Yes	NA
Kasarala G, et al. 2017	Unclear	Yes	Yes	No	Yes	Unclear	Yes	Yes	NA
Koller T, et al. 2017	Yes	Yes	Unclear	Yes	Yes	Unclear	Unclear	Yes	NA
Kumar-Dixit V, et al. 2020	Unclear	Unclear	Unclear	No	Yes	Unclear	Unclear	Yes	NA
Le NHQ, et al. 2014	Unclear	Yes	Unclear	No	Yes	Yes	Yes	Yes	NA
Lee A, et al. 2019	No	Yes	Unclear	No	Yes	Unclear	Unclear	Yes	NA
Lee AS, et al. 2022	Unclear	Yes	No	No	Yes	Yes	Yes	Yes	NA
Li D, et al. 2017	No	Yes	Unclear	Unclear	Yes	Unclear	Yes	Yes	NA
Likhitsup A, et al. 2019	Unclear	Yes	No	Yes	Yes	Unclear	Yes	Yes	NA
Lopes M, et al. 2021	No	Yes	No	No	Yes	Unclear	Yes	Yes	NA
Magri S, et al. 2019	Unclear	Yes	Unclear	Yes	Yes	Yes	Yes	Yes	NA
Mancina RM, et al. 2020	No	Yes	Unclear	Yes	Yes	Yes	Yes	Yes	NA
Martínez-Domínguez SJ, et al. 2022	Yes	Yes	Yes	No	Yes	Yes	Yes	Yes	NA
McHenry S, et al. 2020	Unclear	Yes	Yes	Yes	Yes	Yes	Yes	Yes	NA
Mehrotra P, et al. 2013	Unclear	Yes	No	No	Yes	Unclear	Yes	Yes	NA
Mehrotra P, et al. 2018	Unclear	Yes	Unclear	No	Yes	Unclear	Yes	Yes	NA
Mohammed T, et al. 2012	No	Yes	Unclear	No	Yes	Unclear	Yes	Yes	NA
Monteserin L, et al. 2017	Unclear	Yes	No	No	Yes	Yes	Yes	Yes	NA
Morsy KH, et al. 2012	No	Yes	No	Yes	Yes	Unclear	Yes	Yes	NA
Nasrullayeva F, et al. 2020	Unclear	Unclear	Yes	No	Yes	Unclear	Yes	Yes	NA
Nguyen DL, et al. 2014	Yes	Yes	Yes	Yes	Yes	Unclear	Yes	Yes	NA
Paparo F, et al. 2012	Unclear	Unclear	Yes	Unclear	Yes	Unclear	Unclear	Yes	NA
Patel NP, et al. 2011	Unclear	Yes	Yes	No	Yes	Unclear	Yes	Yes	NA
Perrett AD, et al. 1971	Unclear	Unclear	Yes	Unclear	Yes	Unclear	No	Yes	NA
Perrett AD, et al. 1971	Unclear	Unclear	Yes	Unclear	Yes	Unclear	No	Yes	NA
Principi M, et al. 2018	Yes	Yes	Yes	Yes	Yes	Yes	Yes	Yes	NA
Regner E, et al. 2020	Unclear	Yes	Yes	No	Yes	Unclear	Yes	Yes	NA
Restellini S, et al. 2017	Yes	Yes	Yes	Unclear	Yes	Yes	Yes	Yes	NA
Ritaccio G, et al. 2021	Yes	Yes	Yes	Yes	Yes	Unclear	No	Yes	NA
Rodríguez-Duque JC, et al. 2023	No	Yes	Yes	Yes	Yes	Yes	Yes	Yes	NA
Sagami S, et al. 2017	Unclear	Unclear	Yes	Yes	Yes	Yes	Yes	Yes	NA
Saroli Palumbo C, et al. 2019	Unclear	Yes	Yes	Yes	Yes	Yes	Yes	Yes	NA

Sarmini MT, et al. 2019	Unclear	Unclear	Yes	Unclear	Yes	Unclear	Unclear	Yes	NA
Sarmimi MT, et al. 2019	Unclear	Unclear	Yes	Unclear	Yes	Unclear	Unclear	Yes	NA
Scrivo B, et al. 2021	Yes	Unclear	Yes	Unclear	Yes	Yes	Yes	Yes	NA
Silva J, et al 2019	Yes	Yes	Yes	Yes	Yes	Unclear	Unclear	Yes	NA
Simon TG, et al. 2018	Yes	Yes	Yes	Yes	Yes	Unclear	Yes	Yes	NA
Sourianarayanane A, et al. 2013	Unclear	Yes	Yes	Yes	Yes	Yes	Unclear	Yes	NA
Sousa P, et al. 2021	Yes	Unclear	Unclear	Unclear	Yes	Yes	Yes	Yes	NA
Spagnuolo R, et al. 2019	Unclear	Unclear	No	Yes	Yes	Yes	Yes	Yes	NA
Stoleru G, et al. 2020	Unclear	Unclear	Yes	Unclear	Yes	Unclear	Yes	Yes	NA
Thayumanavan JG, et al. 2010	Unclear	Unclear	No	No	Yes	No	Unclear	Yes	NA
Thin LW, et al. 2012	Yes	Unclear	Unclear	Unclear	Yes	Yes	Yes	Yes	NA
Tirath A, et al. 2019	Unclear	Yes	Unclear	No	Yes	Yes	Yes	Yes	NA
Van Lingen E, et al. 2022	Yes	Yes	Unclear	Yes	Yes	Yes	Yes	Yes	NA
Veltkamp C, et al. 2022	Unclear	Yes	Unclear	Yes	Yes	Yes	Yes	Yes	NA
Voss J, et al. 2021	Yes	Yes	Yes	Yes	Yes	Unclear	Unclear	Yes	NA
Yamamoto-Furusho JK, et al. 2010	Yes	Yes	Yes	Yes	Yes	Unclear	Unclear	Yes	NA
Yen H-H, et al. 2021	Unclear	Yes	Unclear	Yes	Yes	Yes	Yes	Yes	NA
Young S, et al. 2015	Unclear	Unclear	Yes	No	Yes	No	Yes	Yes	NA

Table S5. Pooled prevalence (proportion with 95% confidence intervals) of non-alcoholic fatty liver disease in patients with inflammatory bowel disease according to diagnostic methods used.

Diagnostic Method	n	% (95%Confidence Interval)	I2
Controlled attenuation parameter	17	38.8 (33.1 – 44.7)	90.3
Biopsy after altered liver function tests	4	30.7 (22.8 – 39.1)	47.1
Hepatic Steatosis Index	6	30.4 (26.1 - 35)	66.8
Ultrasounds	28	28.5 (23.1 – 34.2)	97.4
Magnetic resonance imaging	4	25.1 (13.1 – 39.5)	93.8
Fatty Liver Index	2	23.8 (10.9 – 37.8)	-
Computed tomography	5	23.2 (7.6 – 44.3)	98.6
Multiple methods	5	20 (10.8 – 31.3)	98.7
Imaging (with no details)	4	13.8 (9.1 – 19.4)	93.1
Administrative databases	5	13 (4.5 – 25.9)	99.9
No method described	8	11.5 (7.1 – 16.6)	93.3
ICD Codes	6	4 (2.8 – 11.7)	99.9

n, number of studies, ICD: International Classification of Diseases.

Table S6. Pooled prevalence (proportion with 95% confidence intervals) liver fibrosis in patients with inflammatory bowel disease and non-alcoholic fatty liver disease (NAFLD), overall and according to diagnostic methods used.

Diagnostic Method	n	% (95% Confidence Interval)	I^2
Fibrosis overall	20	16.7 (12.2 – 21.7)	88.9
Transient elastography	11	23.6 (17.4 – 30.4)	83.7
Fibrosis-4 (FIB.4) index	6	14.2 (8.2 – 21.4)	83.9
NAFLD Fibrosis Score	2	3.7 (2 – 5.3)	-

n, number of studies; I^2, inconsistency.

Table S7. Number of patients (n) and prevalence of cardiometabolic risk factors in patients with inflammatory bowel disease evaluated for the presence of non-alcoholic fatty liver disease.

Author, year	Disease type	Metabolic factors n (%)								
		Obesity/Over weight n (%)	Type-2 diabetes n (%)	Waist circumference ≥ 90 cm in men and ≥ 80 cm in women	Blood pressure ≥ 130/85 mmHg or requiring specific drug treatment	Triglyceride levels ≥ 150 mg/dL or requiring specific drug treatment	HDL cholesterol levels < 40 mg/ dL for men and < 50 mg/dL for women	Prediabetes (i.e., fasting glucose levels 100–125 mg/dL, or 2-h post-load glucose levels 140 to 199 mg/dL, or HbA1c 5.7%-6.4%)	C-reactive protein (CRP) level > 2 mg/L	Homeostasis model assessment (HOMA) of insulin resistance score ≥ 2.5
Abomhyaa A, et al. 2022	CD	1522 (28.9%)	1501 (28.5%)	-	2186 (41.5%)	1485 (28.2%)	-	-	-	-
Areira C, et al. 2019	IBD	21 (28.8%)	8 (11%)	-	16 (21.9%)	-	-	-	-	-
Bessissow T, et al2016	IBD	3 (2.8%)	9 (8.9%)	-	11 (10.2%)	-	-	-	-	-
Bosch DE, et al 2017	IBD	-	6 (20%)	-	9 (32%)	-	-	-	-	-
Cahill J, et al. 2019	IBD	79 (53.3%)	26 (17.2%)	-	35 (23.3%)	-	-	-	23 (36.5%)	-
Cohen ME, et al. 2021	IBD	2 (40%)	-	-	-	-	-	-	-	-
				-			-		-	16 (59.3%)
Dorobăț A, et al. 2018	IBD	-	-		-		-		-	
Dundulis J, et al. 2014	IBD	-	8 (27.6%)	-	-	-	-	-	-	-
				-		-		-		468 (90%)
Elchert JA, et al. 2018	CD	312 (60%)	270 (52%)		-		-		-	
				-	369 (71%)	-	338 (65%)	-	-	-
					-		-		-	359 (97%)
Elchert JA, et al. 2018	UC	211 (57%)	218 (59%)		-		-		-	
				-	281 (76%)	-	281 (76%)	-	-	-
Glassner K, et al. 2017	IBD	23 (41.1%)	9 (16.1%)	-	19 (33.9%)	-	-	-	-	-
	IBD	-	13 (6.1%)	-	-	-	-	-	-	-
Hoffmann P, et al. 2020	CD	-	7 (4.8%)	-	-	-	-	-	-	-
	UC	-	6 (8.8%)	-	-	-	-	-	-	-
Hong Q, et al. 2022	CD	-	-				29 (34.9%)			
Kang MK, et al. 2020	IBD	11 (22.4%)	5 (10.2%)				18 (36.7%)			
				-	11 (22.5%)	-	-	-	-	-
Kasarala G, et al. 2017	IBD	1246 (22.3%)	-	-	-	-	-	-	-	-
Magri S, et al. 2019	IBD	22 (30.6%)	7 (9.7%)				22 (30.6%)			
McHenry S, et al. 2020	CD	77 (65.3%)	15 (12.7%)	-	45 (38.1%)	-	20 (16.9%)	-	-	-
Principi M, et al. 2018	IBD	-	18 (13.8%)	57 (43.8%)	42 (32.3%)	-	-	-	-	-
	IBD	4 (66.7%)	-	-	-	-	-	-	-	-
Regner E, et al. 2020	CD	3 (75%)	-	-	-	-	-	-	-	-
	UC	1 (50%)	-	-	-	-	-	-	-	-
Ritaccio G, et al. 2021	IBD	121 (58.5%)	-	-	-	-	-	-	-	-
	IBD	-	51 (6.1%)	-	197 (23.7%)	-	-	-	-	-

Rodríguez-Duque JC ,et al. 2023	CD	-	-	-	-	-	-	-	-	-
	UC	-	-	-	-	-	-	-	-	-
Sagami S, et al. 2017	CD	24 (36.4%)	-	-	-	-	-	-	-	-
Saroli Palumbo C, et al. 2019	IBD	-	13 (10.3%)	-	26 (20.6%)	-	-	-	-	-
Sarmini MT, et al. 2019	CD	2030 (48.4%)	1775 (42.4%)	-	-	-	-	-	-	-
	UC	1370 (55.9%)	1320 (53.9%)	-	2021 (82.5%)	-	-	-	-	-
Sourianarayanane A, et al. 2013	IBD	39 (51.3%)	9 (11.8%)	-	21 (27.6%)	-	22 (28.9%)<br>-	-	-	-
Stoleru G, et al. 2020	IBD	-	3 (1.4%)	-	6 (2.8%)	-	-	-	-	-
Tirath A, et al. 2019	CD	-	5 (11.9%)	-	-	-	-	-	-	-
Yen H-H, et al. 2021	IBD	18 (75%)	3 (12.5%)							

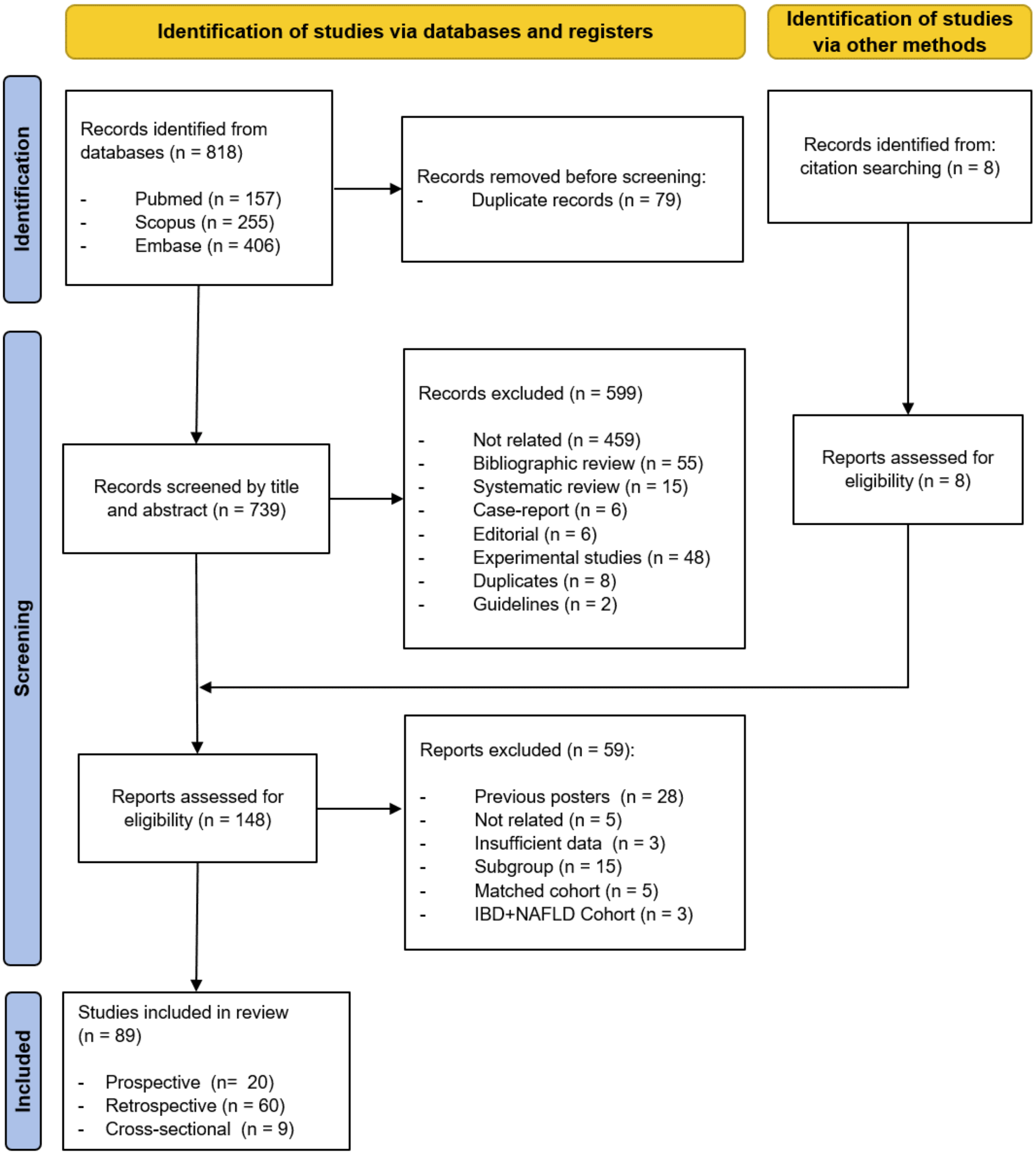

Figure S1. PRISMA flow diagram of study selection process.

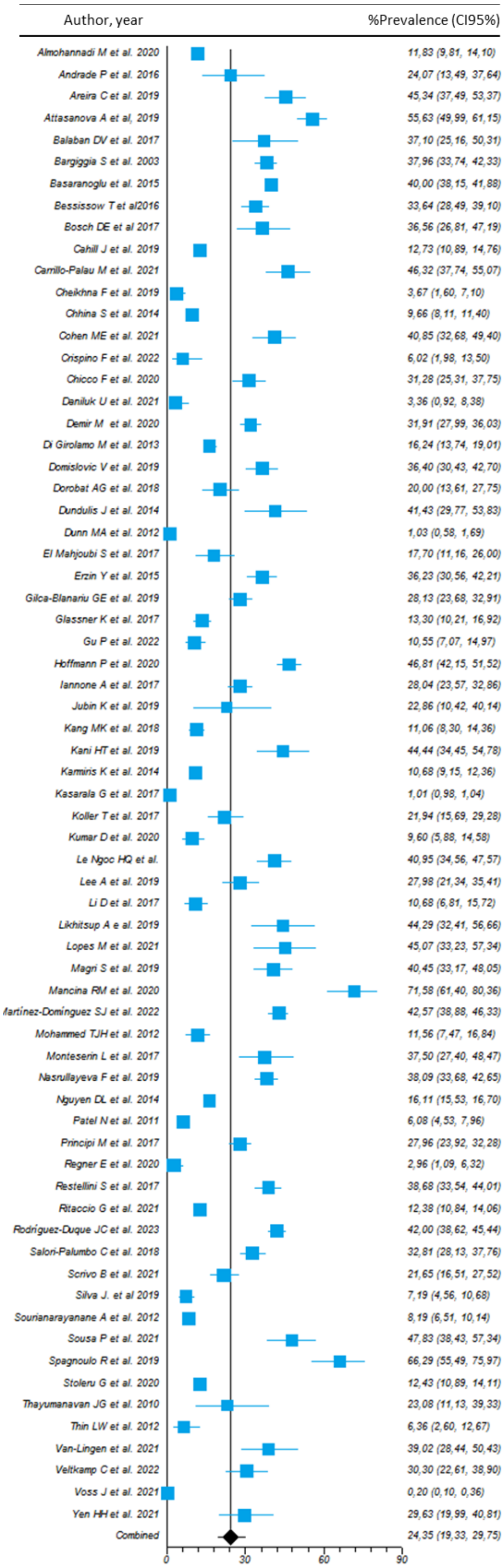

Figure S2. Forest plots of pooled prevalence for nonalcoholic fatty liver disease in patients with inflammatory bowel disease overall.

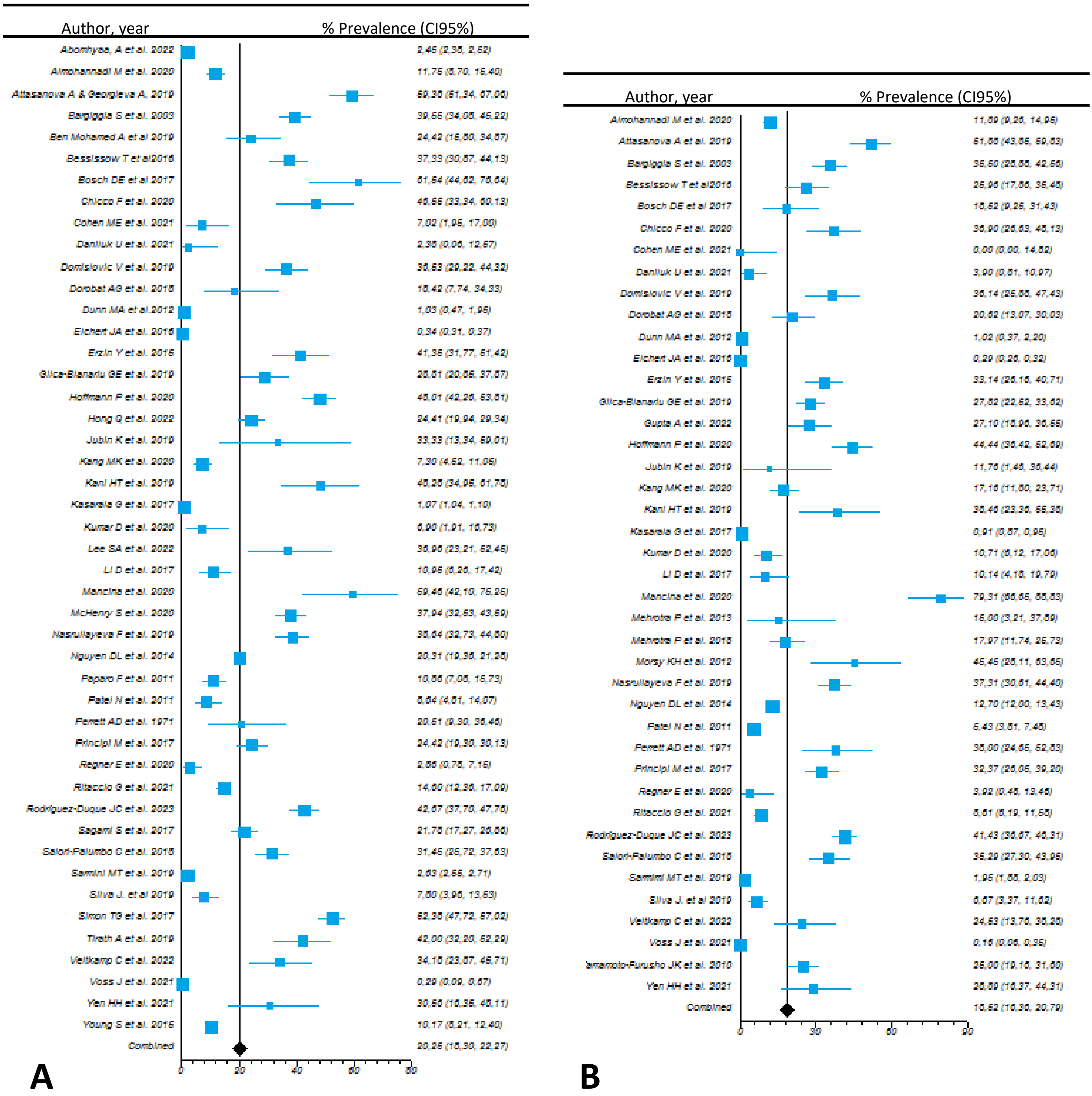

Figure S3. Forest plot of the pooled prevalence of non-alcoholic fatty liver disease in patients with Crohn’s disease (**A**) and ulcerative colitis (**B**).

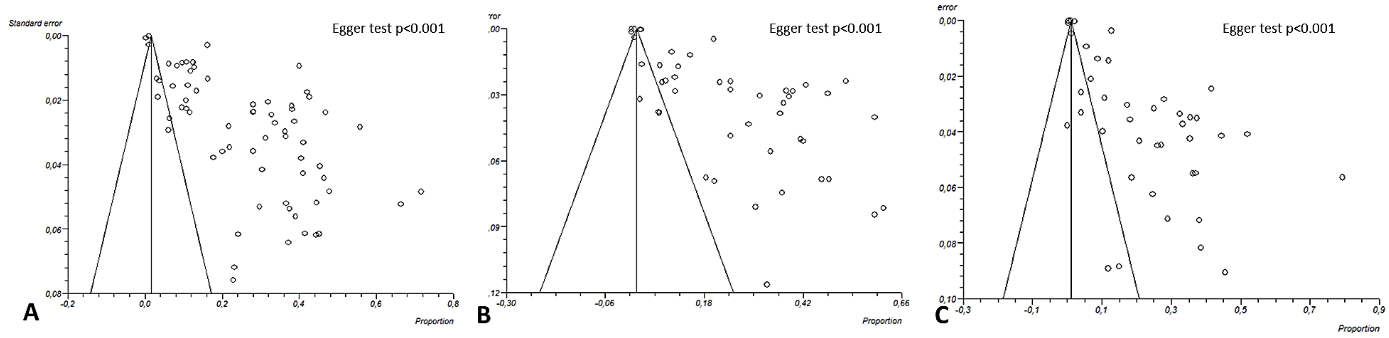

Figure S4. Funnel plot for publication bias of studies reporting on the prevalence of non-alcoholic fatty liver disease in patients with inflammatory bowel disease overall (**A**), Crohn's disease (**B**) and ulcerative colitis (**C**). Significant publication bias was found in Egger and Begg tests.

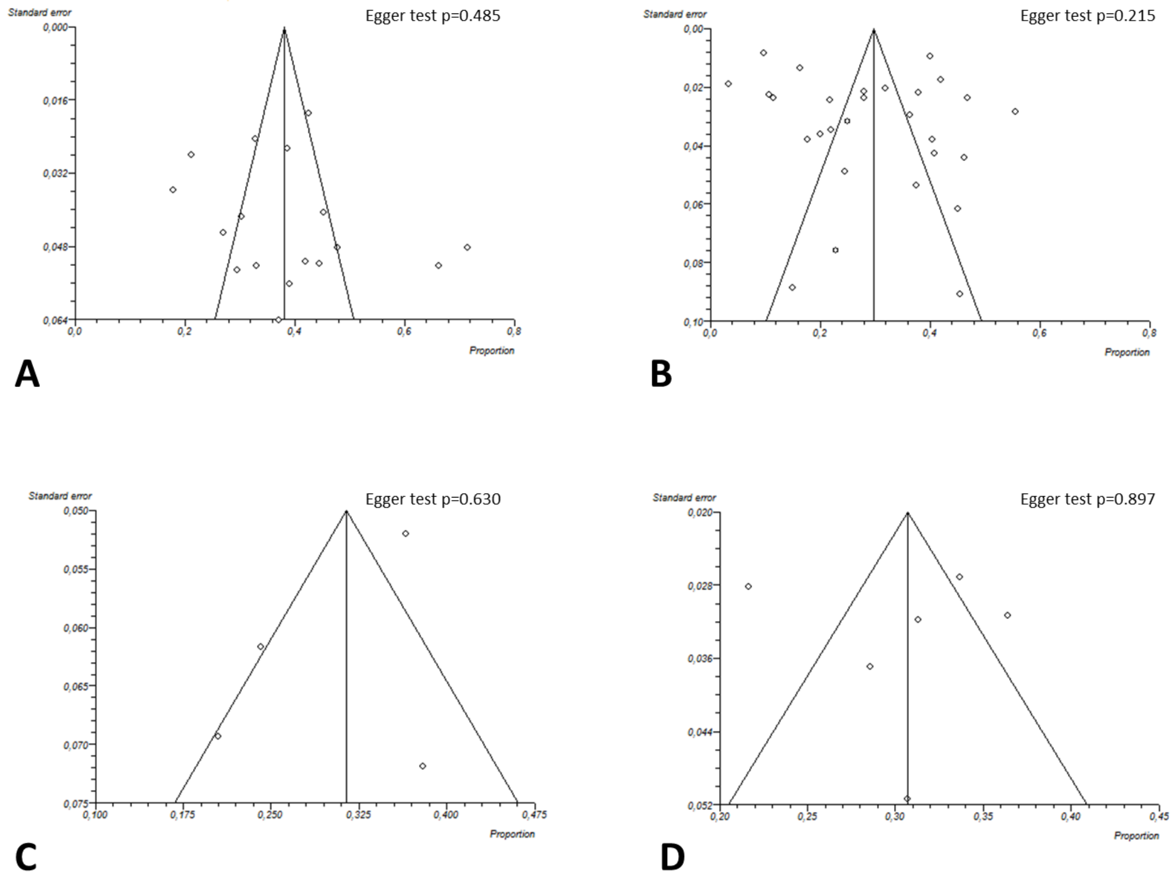

Figure S5. Funnel plots of the studies reporting on the prevalence of non-alcoholic fatty liver disease assessed by (**A**) controlled attenuation parameter (CAP) measure; (**B**) liver ultrasound; (**C**) liver biopsy; and (**D**) hepatitis steatosis index (HIS) score.

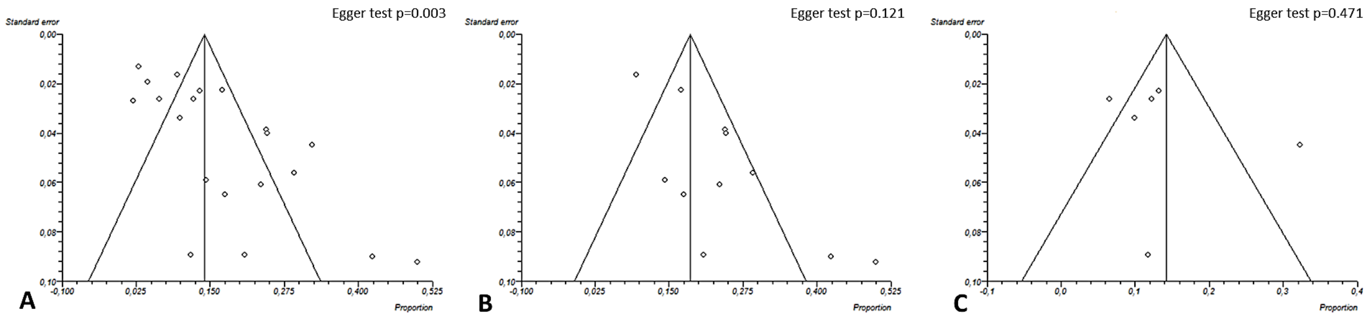

Figure S6. Funnel plots of the studies reporting on fibrosis in patients with inflammatory disease who presented non-alcoholic fatty liver disease, when it was assessed by any method (**A**), transient elastography (**B**) or biochemical indexes (**C**).

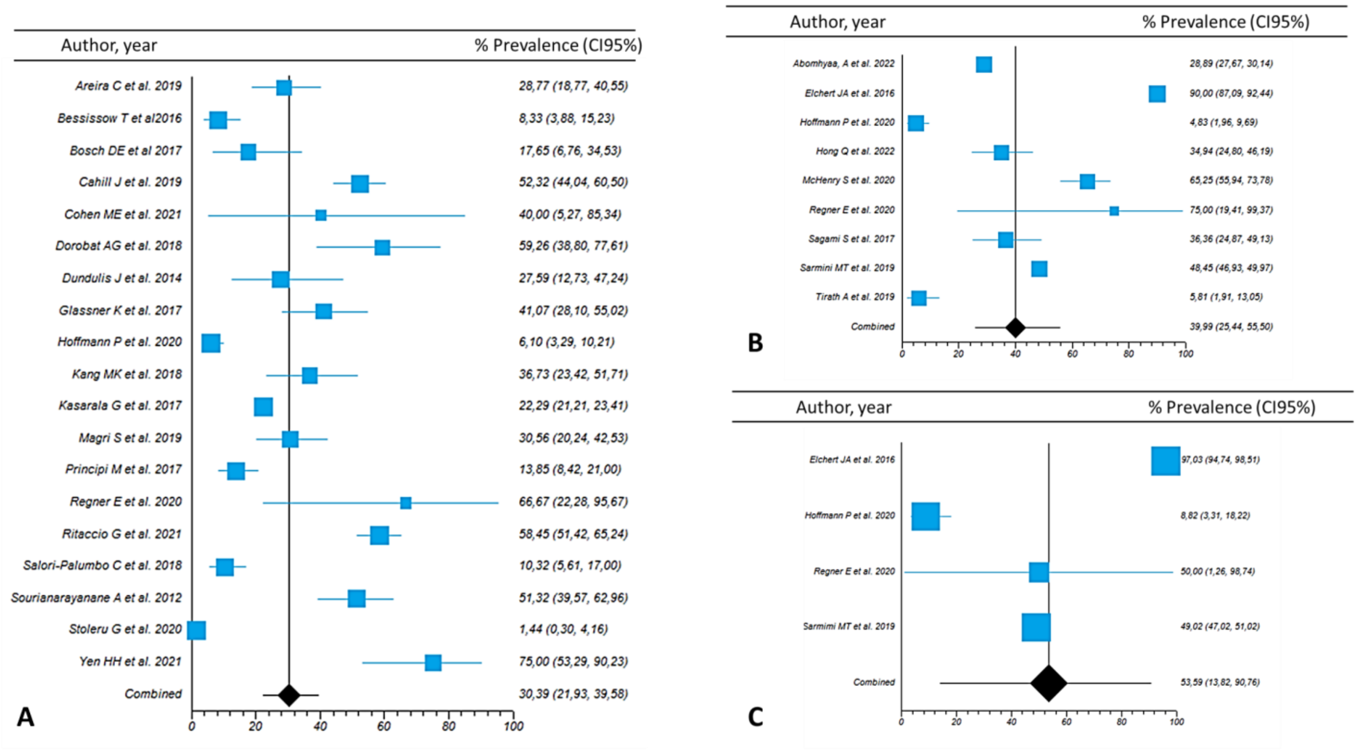

Figure S7. Forest plot of the pooled prevalence of metabolic (dysfunction-) associated fatty liver disease among patients with inflammatory bowel disease overall (**B**), Crohn's disease (**B**) and ulcerative colitis (**C**) who present non-alcoholic fatty liver disease.

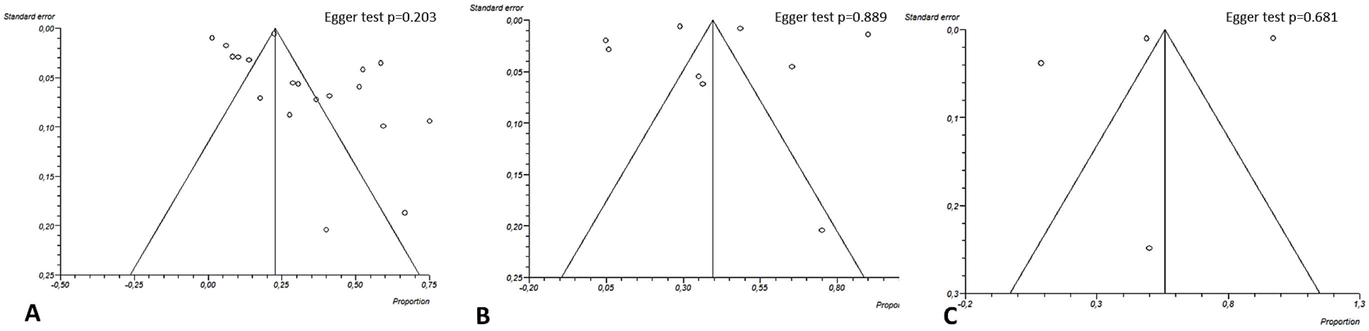

Figure S8. Funnel plots of the studies reporting on the pooled prevalence of metabolic (dysfunction-) associated fatty liver disease among patients with inflammatory bowel disease overall (**B**), Crohn's disease (**B**) and ulcerative colitis (**C**) who present non-alcoholic fatty liver disease.
